# Primary Human Pancreatic Cancer Cells Cultivation in Microfluidic Hydrogel Microcapsules for Drug Evaluation

**DOI:** 10.1002/advs.202206004

**Published:** 2023-02-19

**Authors:** Taiyu Song, Hui Zhang, Zhiqiang Luo, Luoran Shang, Yuanjin Zhao

**Affiliations:** ^1^ Department of Rheumatology and Immunology Institute of Translational Medicine The Affiliated Drum Tower Hospital of Nanjing University Medical School Nanjing 210002 China; ^2^ State Key Laboratory of Bioelectronics School of Biological Science and Medical Engineering Southeast University Nanjing 210096 China; ^3^ Zhongshan‐Xuhui Hospital and the Shanghai Key Laboratory of Medical Epigenetics the International Colaboratory of Medical Epigenetics and Metabolism (Ministry of Science and Technology) Institutes of Biomedical Sciences Fudan University Shanghai 200032 China; ^4^ Chemistry and Biomedicine Innovation Center Nanjing University Nanjing 210023 China

**Keywords:** drug evaluation, hydrogel, microcapsule, microfluidics, pancreatic cancer

## Abstract

Chemotherapy is an essential postoperative treatment for pancreatic cancer, while due to the lack of effective drug evaluation platforms, the therapeutic outcomes are hampered by tumor heterogeneity among individuals. Here, a novel microfluidic encapsulated and integrated primary pancreatic cancer cells platform is proposed for biomimetic tumor 3D cultivation and clinical drug evaluation. These primary cells are encapsulated into hydrogel microcapsules of carboxymethyl cellulose cores and alginate shells based on a microfluidic electrospray technique. Benefiting from the good monodispersity, stability, and precise dimensional controllability of the technology, the encapsulated cells can proliferate rapidly and spontaneously form 3D tumor spheroids with highly uniform size and good cell viability. By integrating these encapsulated tumor spheroids into a microfluidic chip with concentration gradient channels and culture chambers, dynamic and high‐throughput drug evaluation of different chemotherapy regimens could be realized. It is demonstrated that different patient‐derived tumor spheroids show different drug sensitivity on‐chip, which is significantly consistent with the clinical follow‐up study after the operation. The results demonstrate that the microfluidic encapsulated and integrated tumor spheroids platform has great application potential in clinical drug evaluation.

## Graphical abstract

Primary human pancreatic cancer cells encapsulated in hydrogel microcapsules with good biocompatibility and accessibility can proliferate rapidly and spontaneously form 3D tumor spheroids with highly uniform size and good cell viability. Integrating these encapsulated tumor spheroids into a microfluidic chip with a gradient generator and culture chambers enables dynamic and high‐throughput drug evaluation of different chemotherapy regimens.

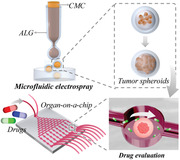

## 1 Introduction

Pancreatic cancer is one of the deadliest malignancies in humans, for which the long‐term survival for these cancer patients remains less than 10%.^[^ ^1^ ^]^ Clinically, surgical resection is regarded as a relatively effective and primary strategy for pancreatic cancer treatment. However, most patients still face an increased risk of recurrence after tumor resection operations.^[^ ^2^ ^]^ Therefore, postoperative adjuvant treatment is usually combined with chemotherapy in clinical practice to prolong the progression‐free survival of patients that undergo surgery.^[^ ^3^ ^]^ At present, various therapeutic drugs, such as gemcitabine (Gem) and fluorouracil, have shown certain efficacy in the clinical treatment of pancreatic cancer.^[^ ^4^ ^]^ Unfortunately, their therapeutic outcomes are hampered by tumor heterogeneity among individuals^[^ ^5^ ^]^ and clinicians can only rely on their clinical experience to apply drug treatments to different patients. Thus, an effective drug evaluation platform is anticipated for simulating pancreatic tumor drug response in vivo to guide clinical medication.

**Figure 1:**
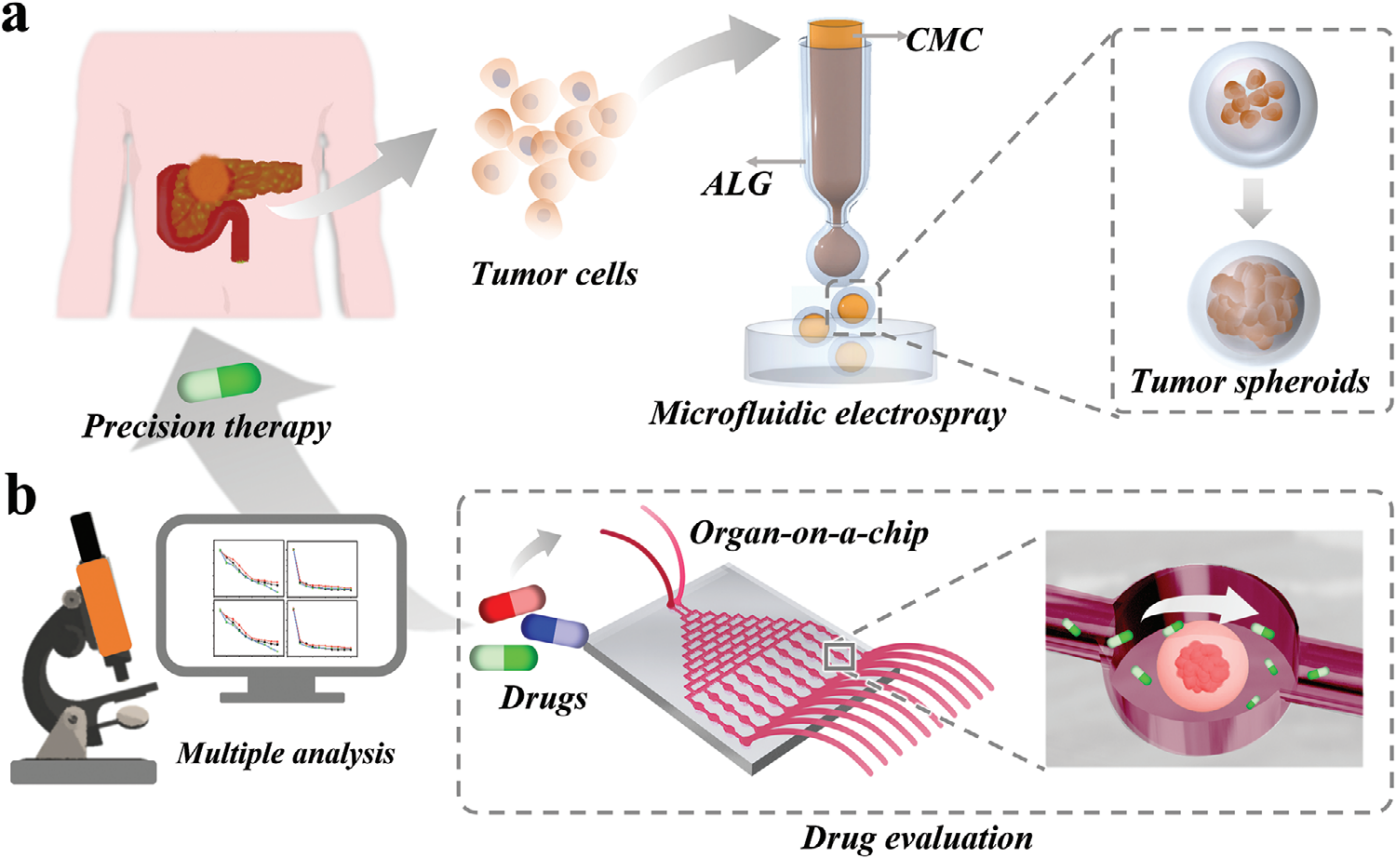
Schematic of tumor peripheral circulation of pancreatic tumor and hydrogel microcapsules integrated microfluidic chip for drug evaluation. a) The design of the tumor spheroids‐encapsulated hydrogel microcapsules. b) The microcapsules‐integrated chip for drug evaluation.

Here, we develop a novel microfluidic hydrogel microcapsule with the ability to encapsulate primary human pancreatic cancer cells and form 3D biomimetic tumor spheroids for drug evaluation, as schemed in **Figure** **1**. 3D tumor spheroids can maintain significant bidirectional interactions between tumor cells and extracellular microenvironment compared with traditional 2D culture systems. Thus, they have been perceived as an accurate tumor model in vitro that can simulate complex cancer microenvironment and have been widely used for drug screening.^[^ ^6^ ^]^ However, the existing tumor spheroids are usually cultured in simple holes, which lack external confinement.^[^ ^7^ ^]^ In addition, the heterogeneity of tumor spheroid size and the difference in tumor spheroids derived from cell lines hinder the possibility of simulating tumor variability across individuals. Moreover, the dynamic supply of nutrients and drug uptake is still challenging to achieve in current 3D tumor models.^[^ ^8^ ^]^ In contrast, microfluidic technologies can precisely manipulate microscale fluids in integrated channels,^[^ ^9^ ^]^ thereby realizing controllable nutrient supply or drug distribution in the systems.^[^ ^10^ ^]^ Also, microfluidics can generate functional microparticles or microcapsules with adjustable and uniform sizes, shapes, and tailored microstructures for cell encapsulation and 3D culture.^[^ ^11^ ^]^ Thus, it is conceived to develop an effective platform with the desired features for drug evaluation by integrating the advantages of 3D tumor models and microfluidic technologies.

For this purpose, we employed a microfluidic electrospray technique to encapsulate primary human pancreatic cancer cells into hydrogel microcapsules for 3D cultivation. The microcapsules were composed of carboxymethyl cellulose (CMC) cores and alginate (ALG) shells, which exhibited high uniformity, stability, and precise dimensional controllability. Both ALG^[^ ^12^ ^]^ and CMC^[^ ^13^ ^]^ have good biocompatibility and accessibility. Besides, compared with photopolymerization, the ionic crosslinking process of sodium alginate is simple and rapid and it has less damage to encapsulated cells. Benefiting from these properties, encapsulated primary pancreatic cancer cells can proliferate rapidly in the semipermeable hydrogel microcapsules and spontaneously form 3D tumor spheroids with highly uniform size and good cell viability. The crosslinked hydrogel showed certain mechanical strength and was not easy to disintegrate, contributing to the long‐term stability of the tumor spheroids necessary for drug screening. In addition, by integrating these encapsulated tumor spheroids into a microfluidic chip with a gradient generator and culture chambers, dynamic and high‐throughput drug evaluation of different chemotherapy regimens could be achieved. It was demonstrated that the encapsulated pancreatic tumor spheroids, derived from different patients, exhibited significant heterogeneity in drug sensitivity to the same treatment. Notably, we have revealed an evident consistency between the data obtained from the encapsulated tumor spheroids and the clinical data obtained from corresponding patients in response to chemotherapy agents. These results indicate that the integration of the 3D tumor models and microfluidic technologies could provide a reliable and accurate drug evaluation platform for the clinical treatment of pancreatic cancer.

## 2 Results and Discussion

**Figure 2:**
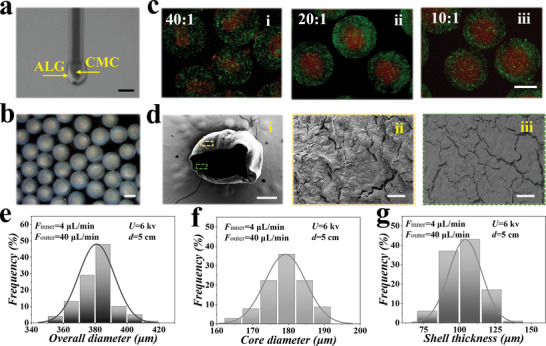
Preparation and regulation of the CMC/ALG microgels. a) The real‐time image showing the generation of CMC/ALG droplets. b) Uniform core–shell hydrogel microcapsules observed under a stereomicroscope. c) Fluorescent images of microcapsules generated under different flow rate ratios. The ALG and CMC precursor solutions were mixed with fluorescent polystyrene nanoparticles L4655 (green) and L3280 (red), respectively. d) SEM images of i) a freeze‐dried microcapsule and ii) the surface of the shell and iii) core. e) Overall size distributions of the CMC/ALG microcapsules under the parameters listed in the chart. f) Core size distributions of the CMC/ALG microcapsules under the parameters listed in the chart. g) Shell thickness distributions of the CMC/ALG microcapsules under the parameters listed in the chart. Scale bars are 500 µm in (a, b), 200 µm in (c), 200 µm in (d,i), and 10 µm in (d,ii, d,iii).

In a typical experiment, we utilized a microfluidic electrospray technique for the one‐step fabrication of hydrogel microcapsules with high homogeneity and biocompatibility. A capillary microfluidic device with a coaxial geometry was designed. An aqueous solution of CMC served as the core flow and another aqueous solution of ALG with high viscosity served as the shell flow. The core and shell flow hydrogel were slowly transfused into the inner and outer channels of the device, respectively, as shown in **Figure** **2**a. The CMC flow was surrounded by the ALG flow at the outlet of the device, where the two flows were subjected to electrospray and formed stable core–shell droplets. Then, the shell of the hydrogel droplets was solidified through reacting with calcium chloride in the collection pool and CMC/ALG microcapsules were generated continuously, as presented in Figure 2b. The fast ionic crosslinking process ensured that the inner CMC hydrogel was restrained inside the microcapsules, which provided a confined space for subsequent cell cultivation. We also found that 1.5 wt% sodium alginate could produce a suitable viscosity difference with the internal carboxymethyl cellulose (1.0 wt%) phase and this ensured a good core–shell structure, as presented in Figure S1 in the Supporting Information.

To generate microcapsules with uniform and controllable sizes, we explored multiple key parameters. We found that when keeping the outer phase rate constant and increasing the inner phase rate from 1 to 6 µL min^−1^, the diameters of both capsules and the inner core increased markedly, as recorded in Figure S2a in the Supporting Information. Besides, when the inner flow rate was set constant, the volume of the microcapsules expanded when the outer flow rate increased from 30 to 55 µL min^−1^, while the core diameter showed the opposite tendency (Figure S2b, Supporting Information). Moreover, the voltage and the distance (between the device outlet and the collection) can also affect the volume of the capsules, as shown in Figure S2c,d in the Supporting Information. Considering that a high voltage can damage cells, we selected 6 kv as the final voltage during the electrospray process, under which microcapsules with stable core–shell structure can be prepared. To visualize the different shell thickness of the microcapsules generated under different outer/inner flow rate ratios, red and green fluorescent nanoparticles were utilized to label the core and shell, respectively, as shown in Figure 2c. It was found that the red fluorescent nanoparticles stayed in the microcapsule interior after 11 d, which revealed that carboxymethyl cellulose was maintained in the core during culture. Furthermore, a single freeze‐dried microcapsule was characterized with a scanning electron microscopy (SEM), which validated the core–shell structure, as shown in Figure 2d. With that, the microcapsule size could be finely tuned and the monodispersity of the microcapsules could be ensured (Figure 2e–g). Besides, the porous structures of the shell hydrogel were confirmed by SEM imaging, which suggested that the microcapsules could support the entry of nutrients and elimination of metabolic waste, as shown in Figure S3 in the Supporting Information.

**Figure 3:**
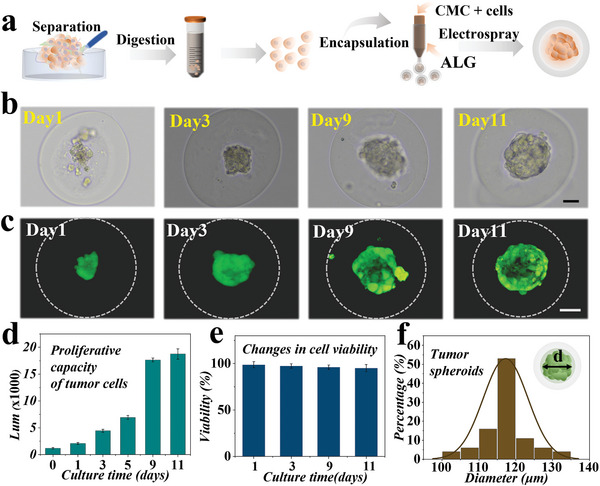
Generation and characterization of tumor spheroids encapsulated in the hydrogel microcapsules. a) Schematic of the extraction of primary pancreatic cancer cells and the preparation of cell‐laden microcapsules through microfluidic electrospray. b) Bright‐field images of primary pancreatic tumor cells after encapsulation in the microcapsules at 1, 3, 9, and 11 d. c) Live/dead cell staining of tumor spheroids in the microcapsules at days 1, 3, 9, and 11. d) Quantitative analysis of cell proliferation of tumor spheroids at days 1, 3, 5, 9, and 11 through CellTiter‐Glo 3D cell viability assay. Lum refers to the luminescent signal indirectly reflecting the amount of adenosine triphosphate (ATP) and the number of cells (*n* = 6 for each group). e) Quantitative analysis of cell viability of tumor spheroids at days 1, 3, 9, and 11 (*n* = 6 for each group). f) Size distribution of tumor spheroids at day 11 (*n* = 100 for each group). Data are shown as mean ± SD. Scale bars are 50 µm in (b, c).

We then explored the feasibility of the microcapsules as 3D carriers for the formation of pancreatic tumor spheroids from primary pancreatic cancer cells. Tumor tissue derived from pancreatic cancer patients undergoing surgical resection was cut into small pieces and isolated into cell suspension using Type IV collagenase, as exhibited in Figure S4 in the Supporting Information. The tumor cell suspension was mixed with the CMC solution to obtain cell‐laden microcapsules by electrospray following the experimental steps described above, as schemed in **Figure** **3**a. Then, the primary pancreatic cancer cells encapsulated in the microcapsules were monitored in a cell culture medium for 11 d. On the second day, the cells tended to self‐organize into small clusters in the core of the microcapsules. Over the next few days, the cells showed good viability; the clusters increased in size and finally developed into a well‐defined spheroid, as shown in Figure 3b. Besides, live/dead staining and quantitative analysis results showed an increased cell proliferation and high survival rate during the whole cultivation, confirming excellent biocompatibility of the hydrogel microcapsules, as shown in Figure 3c–e. This fact also demonstrated that primary cells had good viability and maintained proliferation and differentiation abilities even after undergoing the microfluidic electrospray process at 6 kv voltage. Additionally, we evaluated the spheroids' size homogeneity on day 11. The result indicated that the tumor spheroids cultured in the microcapsules had uniform size, as exhibited in Figure 3f. It was worth mentioning that cancer cells isolated from tumor spheroids by the moderate cell‐dissociating enzyme (TrypLE Express Enzyme) could be encapsulated into microcapsules and form a well‐defined spheroid again (Figure S5, Supporting Information). This result suggested that the construction of patient‐derived tumor spheroids in the core–shell microcapsules was scalable.

**Figure 4:**
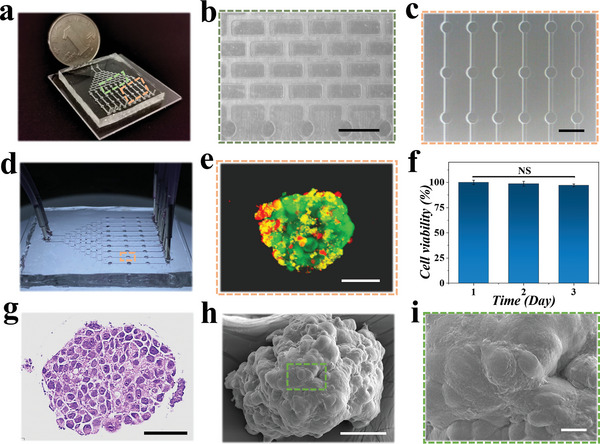
a) Photograph of the microfluidic concentration gradient generator. b) Optical microscopic picture of the chip gradient generator. c) Light microscopy image of the spheroids culture chamber. d) Optical images of the tumor spheroids‐integrated microfluidic chip. e) Confocal laser‐scanning image of a microcapsule‐encapsulated tumor spheroid in the culture chamber with live/dead cell staining. f) Quantitative analysis of cell viability of the tumor spheroids at days 1, 2, and 3 after seeded into the cell culture chamber (*n* = 30 for each group). g) HE staining image of a pancreatic tumor spheroid. SEM images of h) a whole pancreatic tumor spheroid and i) the magnified image showing the surface structure of the tumor spheroid. Scale bars represent 2 mm in (b, c), 50 µm in (e), 50 µm in (g), 50 µm in (h), and 5 µm in (i).

To better mimic drug‐releasing in vivo and achieve high‐throughput drug evaluation, a microfluidic chip with a tree‐shaped fluidic network was constructed as a concentration gradient generator, as recorded in **Figure** **4**a. Specifically, the microfluidic concentration gradient generator was constituted of eight stages of branched channels (Figure 4b), with an incremental number of branches from three to ten. An array of cell culture compartments connecting the terminal branches were used to accommodate tumor spheroid‐laden microcapsules, as shown in Figure 4c. The microcapsules were placed into the culture chamber of the chip and then the chip was sealed. After that, the pancreatic cancer organoid culture medium slowly entered from the inlets of the chip and flowed through the channels to reach the culture chamber, and then flowed out from the outlet of the chip. We first tested the capability of the chip to support cell growth by placing one spheroid‐laden microcapsule in each compartment. Cell culture medium was pumped into the two inlets of the chip by syringe pumps (Figure 4d) and we found that the cells showed satisfactory viability during 3 d of cultivation (Figure 4e,f). During the process of culture and drug screening, the culture medium was always in a dynamic flow process, so that oxygen and nutrients could be continuously transported to the interior of the microcapsule, and the metabolic waste from tumor spheroids could be carried away by the flowing liquid. Notably, the surface characteristics and structure in a single tumor spheroid were validated through hematoxylin‐eosin (HE) staining and SEM imaging, as demonstrated in Figure 4g–i.

**Figure 5:**
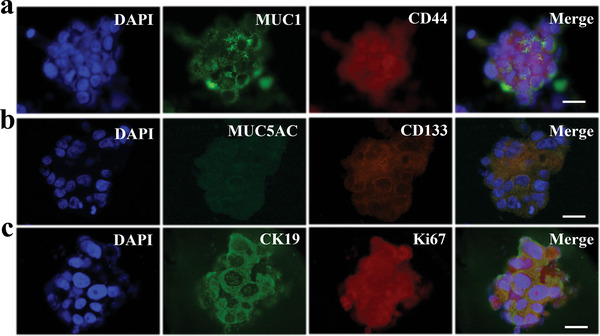
a) Immunofluorescent images of pancreatic tumor spheroid for CD44 and MUC1. b) Immunofluorescent images of pancreatic tumor spheroid for MUC5AC and CD133. c) The fluorescence images of tumor spheroids staining with Ki67 and CK19. a–c) Scale bars are 20 µm.

We tried to simulate real pancreatic cancer from the extracellular matrix components and the stressful environment of tumor cells facing, and verified this simulated effect at the histological level (through Immunofluorescence and Immunohistochemistry).To investigate relative histopathological features of the tumor spheroids, immunohistochemistry for CD44, CD133, MUC1, and MUC5AC in the primary tissues was first conducted, and then the corresponding spheroids were stained by immunofluorescence for the same markers. CD44 and CD133 are often used as cancer stem cell markers in pancreatic cancer. As mucin family members, MUC1 and MUC5AC are often used as mucin markers for the pathological diagnosis of pancreatic cancer. The staining results showed that the tumor spheroids and the corresponding tumor tissues had similar localization and expression in these molecular markers, as exhibited in **Figure** **5**a,b and Figure S6 in the Supporting Information. In addition, the expression of CK19 is associated with pancreatic ductal epithelial cells in human tumors and the high proportion of proliferation marker (Ki67) positive cells indicated the effective proliferation of tumor cells, as indicated in Figure 5c.

**Figure 6:**
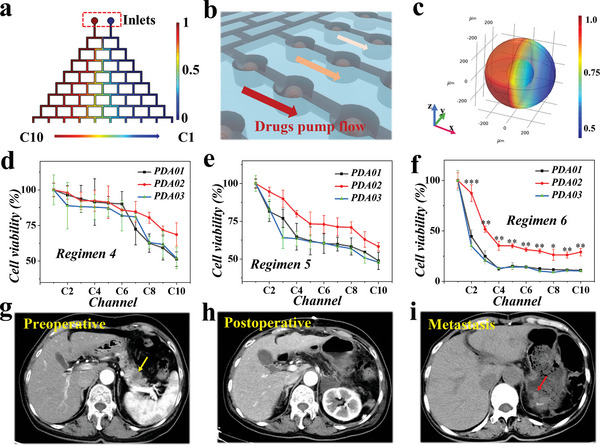
Response of pancreatic tumor spheroids to antineoplastic agents. a) Numerical simulation of the drug concentration gradient formed in the microfluidic concentration gradient generator. b) Schematic of the drug solution flowing through the cell culture chamber. c) Numerical simulation of drug permeating into a microcapsule under the flow (*x*‐axis was the direction of fluid flow). The unit of scale bar was mol m^−3^ (in (a) and (c)). Response of three patient‐derived tumor spheroids to d) 4 h Gem treatment, e) 48 h S‐1 treatment, and f) 4 h GS (combination of Gem and S‐1) and 44 h S‐1 treatment on‐chip (*n* = 30 for each group). There was no statistical difference between PDA01 and PDA03 in regimen 6. Computed tomography data of the PDA02 patient in the g) preoperative, h) postoperative, and i) tumor metastasis (at the fifth chemotherapy cycle) stage.

Whether the antitumor drugs can play a role in suppressing tumor progression mainly depends on the appropriate blood drug concentration. By setting the drug concentration gradient, we can find the appropriate drug concentration that shows the killing effect on the tumor cells while reducing the damage to normal cells. We then conducted a numerical simulation to study the concentration gradient established in the branched channel based on the Navier–Stokes equation.^[^ ^14^ ^]^ The concentration of a simulated drug molecule of the left and right inlets were defined as 1 and 0, respectively, excellent drug concentration gradient was formed (C10 to C1), as shown in **Figure** **6**a and Figure S7a in the Supporting Information. Meanwhile, we simulated the permeation of small molecules across the microcapsules into the tumor spheroids under constant fluid flow based on Fick law.^[^ ^15^ ^]^ The numerical simulation results suggested that relatively high drug concentrations could be obtained in tumor spheroids, as shown in Figure 6c and Figure S7b in the Supporting Information. Besides, Rhodamine B was chosen as the model molecule to visualize the concentration gradient generated in the microfluidic chip, as shown in Figure S8a,b in the Supporting Information. It was found that the fluorescence intensity among terminal branches showed significant difference, forming a concentration gradient consistent with the simulation results. These results demonstrated that the constructed microfluidic device could establish a concentration gradient of chemicals, and thus be suitable for drug screening.

To explore the value of the tumor‐spheroid‐laden microfluidic chip in in vitro drug evaluation, we obtained tumor spheroids derived from three pancreatic cancer patients, named as PDA01, PDA02, and PDA03, respectively. In detail, we placed a tumor spheroid‐encapsulated microcapsule in each culture chamber, and the whole chip hold 30 microcapsules. The tumor spheroids were continuously grown in microcapsules for 9 d prior to drug treatment on‐chip. Besides, the cell apoptosis level of the three tumor spheroids was similar, as quantified by flow cytometry (Figure S9, Supporting Information). Next, we tested the drug evaluation capability of the microfluidic platform. The three tumor spheroids were exposed to Gem (1 µm) and a combination of 5‐FU (Fluorouracil), gimeracil, and oteracil potassium (hereafter named S‐1, 1 µm) for different treatment options, as schemed in Figure S10 in the Supporting Information. When exposed to a constant flow of the drug solution for 72 h (regimen 1, 2, or 3, respectively), the three patients‐derived spheroids exhibited insignificant difference in response to these drugs regimen (*p* > 0.05), as exhibited in Figure S11 in the Supporting Information. Meanwhile, when exposed to a 4 h gem (regimen 4) or 48 h S‐1 (regimen 5) solution, no significant differences were observed among the three tumor spheroids (*p* > 0.05), as shown in Figure 6d, e. However, when the tumor spheroids were exposed to pulsed doses of Gem and S‐1 (regimen 6, in a way similar to clinical therapeutic regimens^[^ ^16^ ^]^), PDA02 showed higher resistance to combination (GS, gem, and S‐1) chemotherapy than the other two tumor spheroids (Figure 6f). Notably, through setting these drug concentration gradients, the dose‐response curves of tumor spheroids derived from different patients can be obtained and IC50 can be calculated to more intuitively compare the difference in drug sensitivity of different individual patient‐derived tumor spheroids. Significantly, a long‐term clinical follow‐up study after the operation showed that the PDA02 patient had multiple metastases after five cycles of Gem and S‐1 chemotherapy, while no metastases were found in Patients PDA01 and PDA03 after eight cycles of chemotherapy, as exhibited in Figure 6g–i. This result showed that these tumor spheroids models could well preserve the heterogeneity of the primary tumor. Actually, the PDA02 patient had poorly differentiated carcinoma, resulting in a higher degree of malignancy, compared with PDA01 and PDA03 patients. Thus, PDA02 patient was more likely to develop drug resistance and show tumor metastasis earlier (**Table** **1**). These results suggested that the combination of tumor spheroids and microfluidic concentration gradient generator could reflect the clinical course of an individual patient. Although with a relatively small sample size, the performance of the proposed microfluidic hydrogel microcapsules‐integrated chip system can be demonstrated to a certain extent. Future study is going to be carried out with a larger sample size and more compelling statistical analysis.

**Table 1:** Clinical information for patients with pancreatic cancer corresponding to the three kinds of tumor spheroids

Patient	Tumor site	Pathology	Differentiation	Chemotherapy
PDA01	Head^a)^	Ductal adenocarcinoma	Moderately	Gem+S‐1
PDA02	Head	Ductal adenocarcinoma	Poorly	Gem+S‐1
PDA03	Head	Ductal adenocarcinoma	Moderately	Gem+S‐1

^a)^ Head: The head of the pancreas.

In this paper, a novel pancreatic cancer model was constructed by using microencapsulation and stem cell culture techniques. The tumor model can fully simulate pancreatic cancer cells proliferation and differentiation as well as their interactions with the extracellular matrix. By combining microencapsulated pancreatic cancer tumor spheroids with microfluidic chips, the nutrient transport and drug penetration and distribution in the tumor microenvironment can be effectively replicated, which makes the tumor model closer to the real tumor microenvironment, thereby improving the accuracy of drug evaluation results. However, our tumor model still lacks some important stromal cells^[^ ^17^ ^]^ and immune cells.^[^ ^18^ ^]^ Relevant research has reported that these components can affect the sensitivity of tumor cells to chemotherapeutic drugs.^[^ ^19^ ^]^ Therefore, we will further analyze the tumor microenvironment of pancreatic cancer to construct an engineered tumor model. In addition, we currently have limited data on drug evaluation for pancreatic cancer, and more tumor samples and relevant data need to be collected, aiming to provide more valuable references for clinical practice.

## 3 Conclusion

In summary, we have fabricated hydrogel microcapsules with a CMC core and ALG shell through the microfluidic electrospray technique for 3D tumor cell cultivation. The hydrogel microcapsules were endowed with uniform and tunable size by regulating the fluid flow rates, voltage, and collection distance. The encapsulated primary pancreatic cancer cells proliferated rapidly in the microcapsules and formed 3D tumor spheroids with highly uniform size and excellent cell viability. Notably, a microfluidic chip with a tree‐like branched channel geometry was constructed with the tumor spheroids integrated into culture chambers. Through this, a concentration gradient of drugs was established for dynamic and high‐throughput screening of different chemotherapy regimens. On‐chip experiments revealed that pancreatic tumor spheroids derived from different patients showed heterogeneous drug sensitivities to the same agent, in a way consistent with clinical data obtained from corresponding patients after the operation. These results indicated that the present microfluidic‐integrated tumor spheroid platform is promising for clinical drug evaluation and could shed light on personalized therapy.

## 4 Experimental Section

### Materials

Alginate and calcium chloride powder were bought from Alfa Aesar. CMC (low viscosity) was purchased from Macklin. Fluorescent polystyrene nanoparticles L4655 and L3280 were obtained from ThermoFisher. Gem, 5‐FU, gimeracil, and oteracil potassium were obtained from MedChemExpress (MCE) and formulated as drug solutions, respectively. Capillaries were purchased from Shanghai Great Wall Scientific Instrument Shop. Alginase was purchased from Sigma. A live/dead staining kit was purchased from KeyGEN BioTECH. Cell culture plates were obtained from Nest Life Science Technology Co., Ltd. Celltiter‐Glo kit was purchased from Promega, USA. Glutaraldehyde and anhydrous ethanol were purchased from Shanghai Hushi Co., Ltd. The primary antibody was provided by Servicebio (WuHan, CHINA). The secondary antibody was provided by Thermo Fisher Scientific. Collagenase II was obtained from Gibco and DNase I was purchased from Roche. TrypLE was purchased from Gibco.

### Preparation of Hydrogel Microcapsules

A microfluidic electrospray device was assembled for preparing core–shell microcapsules. In brief, the inner diameters of two different sizes of capillaries were ≈300 and 100 µm. The microfluidic device was constructed with two cylindrical capillaries on a glass slide adhered at the connection spot with transparent epoxy resin. 1 wt% CMC was used as the internal phase and 1.5 wt% alginate (highly viscous) was used as the external phase. A voltage power supply was applied to generate an electric field between the chip and the collecting pool. The outer ALG sheathed the inner CMC and segregated it into microdroplets through the electrostatic interaction. The voltage, collection distance, and outer/inner phase flow rate were adjusted to fabricate different morphology of microcapsules.

### Human Pancreatic Tumor Specimens

The cancer tissues were isolated from three surgical resection specimens obtained from pancreatic cancer patients at the department of Pancreatic Surgery, Drum Tower Hospital of Nanjing University Medical School (Nanjing, China). Prior to surgery, all patients have signed a written informed consent. All studies were performed according to recognized ethical guidelines (2020‐072‐01) approved by the Ethics Committee of the Affiliated Drum Tower Hospital, Medical School of Nanjing University. The samples were confirmed to be tumors based on histopathological assessment.

### Extraction of Human Pancreatic Tumor Cells

The human pancreatic tissue was minced and digested with collagenase II in a laboratory shaker for a maximum of 1–3 h. The small cell clumps were further isolated with TrypLE enzyme for 15 min at 37 °C. Suspension cells were centrifuged at 1400 rpm/5 min at 4 °C to remove the supernatant. Finally, primary cells were resuspended and washed with cold phosphate buffered saline (PBS), collected in a cell filter.

### Fabrication of Cell‐Laden Microcapsules

The primary tumor cells were resuspended in a 1.0% w/v carboxymethyl cellulose sodium solution. Then, the cell resuspension was served as the inner phase and pumped into the inner capillary of the device with flow rate of 40 µL min^−1^. Meanwhile, the ALG hydrogel was pumped into outer channel with flow rate of 40 µL min^−1^. These resultant cell‐laden capsules were moved to a cell‐culture well‐containing culture medium described below and then incubated in an incubator.

### Pancreatic Tumor Spheroids Culture

The primary pancreatic cancer cells encapsulated in microcapsules were grown in cell culture medium (advanced dulbecco's modified eagle medium/nutrient mixture F‐12 (DMEM/F12), containing Wnt3A media (50%), R‐spondin1 media (10%), hEGF 50 ng mL^−1^, Noggin 100 ng mL^−1^, hFGF10 100 ng mL^−1^, B27 supplement 1%, Glutamax 1%, N‐2‐hydroxyethylpiperazine‐N‐2‐ethane sulfonic acid (HEPES) 10 mm, A83‐01 500 nm, hGastrinI 0.01 µm, *N*‐acetylcysteine 1.25 mm, PGE2 1 µm, and nicotinamide 10 mm). Tumor cells were grown and observed in microcapsules for 11 d, with cell medium refreshed every 2–3 d.

### Cell Viability

The viability of primary cells encapsulated in hydrogel microcapsules was evaluated with continuous culture by using a Live/Dead Kit. The microcapsules encapsulating cancer cells were taken from the culture medium and washed with normal saline solution. Then, these cell‐laden microcapsules were incubated with the Live/Dead dye staining kit and kept in darkness at 37 °C for 30 min, stained for green (live cells) or red (dead cells). For the cell viability quantitative analysis, multicellular clumps cultured in the microcapsules were transferred to 96‐well plates, and an equal volume of cell viability assay solution (CellTiter‐Glo) was added to the well plates and mixed for 10 min at the shaking table. The luminescence (RLUs) was detected using a multifunctional microplate reader with the program set for luminescence (Integration: 500).

### Immunohistochemistry of Human Pancreatic Tumor Tissues

Tissue sections were fixed by formalin, embedded in paraffin, deparaffinized, and then antigen retrieval was performed. An immunohistochemical pen was used to draw circles in sections. Then, the sections were processed with immunohistochemistry staining with MUC1 (ab109185, 1:250), MUC5AC (MA5‐12178, 1:100), CD44 (ab254530, 1:4000), and CD133 (AF06634, 1:500).

### Immunocytochemistry of Pancreas Tumor Spheroids

The tumor spheroids were fixed for 2 h with formalin, improved the permeability of cell membrane with 0.2% Triton‐X100, and blocked with normal goat serum for 1 h. All types of antibodies were diluted with goat serum and incubated with the tumor spheroids overnight and the spheroids were washed three times with cold PBS. Tumor spheroids were mixed with fluorescent‐labeled antibodies and incubation for 1 h, and then mixed with 10 µg mL^−1^ 4',6‐diamidino‐2‐phenylindole (DAPI) for 10 min. Antibodies diluted in goat serum solution included MUC1 (ab109185, 1:250), Ki67 (27309‐1‐AP, 1:200), CD44 (ab254530, 1:250), MUC5AC (MA5‐12178, 1:100), CK19 (10712‐1‐AP, 1:200), and CD133 (AF06634, 1:200). Fluorescent‐labeled antibodies were antimouse IgG (Invitrogen, Alexa‐488, 1:200) and antirabbit IgG (Invitrogen, Alexa‐594, 1:200).

### Apoptosis Detection of the Tumor Spheroids

The microcapsules were first completely dissolved by alginate lyase, and then the tumor spheroids were dissociated into cell suspensions through enzymatic digestion and were washed with cold PBS three times. After centrifugation to discard the supernatant, 500 µL Binding Buffer, Propidium iodide (5 µL) and Fluorescein Isothiocyanate (FITC) Annexin V (5 µL) were mixed, then added to the bottom for resuspending cell pellet. These suspensions were incubated and protected from light for 30 min at room temperature. Relative apoptosis levels were measured by flow cytometry and analyzed by FlowJo VX.

### Characterization

The microstructure of the microcapsules was freeze‐dried and observed through the SEM (AIS‐2100, Navo Nano) after coating with gold‐palladium. Tumor spheroids isolated from the microcapsules were fixed with 4% paraformaldehyde and 2.5% glutaraldehyde and were then dehydrated in gradient ethanol. Sputter‐coating with gold palladium was performed before SEM imaging. Fluorescence imaging of the cell spheroids was carried out by a confocal laser scanning microscope (SZX‐16, Olympus). The 3D composite image of cell spheroids was processed and analyzed by ImarisViewerx64.

### Microfluidic Concentration Gradient Chip Design

The polydimethylsiloxane chip was fabricated using standard soft lithography techniques. The microfluidic chip was composed of two liquid inlets, a concentration gradient generator, cylindrical chambers, and ten liquid outflow tracts. For the concentration gradient generator, straight channels were spatially arranged with an orderly T outline for fluid flows and molecular diffusion. The ten terminal branch channel outlets were further connected with ten arrays of cylindrical chambers, each of which contains three chambers in a row.

### Microfluidic Drug Evaluation

Primary pancreatic cancer cells were cultured off‐chip for 9 d and were then transferred into the microfluidics chip. Each chamber contained one spheroid‐laden microcapsule. Six treatment regimens were set. For regimen 1, drug solution (Gem 1 µm) was pumped into the left channel inlet and the normal cell medium was pumped into the right channel inlet for 72 h. For regimen 2, drug solution (S‐1 1 µm) was pumped into the left channel inlet and the normal cell medium was pumped into the right channel inlet for 72 h. For regimen 3, the drug mixture solution (Gem and S‐1,1 µm, respectively) was pumped into the left channel inlet and the normal cell medium was pumped into the right channel inlet continued for 72 h. For regimen 4, Gem (1 µm) was pumped into the left channel inlet and normal cell medium was pumped into the right channel inlet for 4 h, then Gem was replaced by normal cell medium, which was pumped from both inlets of the channel for another 68 h. For regimen 5, S‐1 (1 µm) was pumped into the left channel inlet and normal cell medium was pumped into the right channel inlet for 48 h, then S‐1 was replaced by normal cell medium, which was pumped from both inlets of the channel for another 24 h. For regimen 6, drug mixture solution (Gem and S‐1,1 µm, respectively) was pumped into the left channel inlet and normal cell medium was pumped into the right channel inlet for 4 h, then the mixture drug was replaced by S‐1 solution (1 µm) and was pumped into the left channel inlet for 44 h, and finally, the S‐1 solution was replaced by normal cell medium, which was pumped from both inlets of the channel for another 24 h. After drug treatment for 72 h, tumor spheroids encapsulated in microcapsules were taken out and a celltiter‐glo assay kit was applied to evaluate the inhibitory effect of chemotherapy regimens.

### Statistical Analyses

All the presented data were normalized on the basis of the control group. All data were shown as means ± standard deviation (SD). Statistical analyses were performed with the student's *t*‐test. The sample size (*n*) was indicated in the figure legends. NS reflects no significant difference. Statistical difference is pointed as * *p* < 0.05, ** *p* < 0.01, or *** *p* < 0.001. Statistical results are shown in the figure legends.

## Conflict of Interest

The authors declare no conflict of interest.

## Supporting information

Supporting Information

## Data Availability

The data that support the findings of this study are available from the corresponding author upon reasonable request.
