# Supplementary material for: Primary Human Pancreatic Cancer Cells Cultivation in Microfluidic Hydrogel Microcapsules for Drug Evaluation
Source: Adv Sci (Weinh). 2023 Feb 19;10(12):2206004. doi: 10.1002/advs.202206004 (PMC10131826; doi:10.1002/advs.202206004)
Supplement: Supplementary file 1 — Supporting Information [file ADVS-10-2206004-s001.pdf]

Supporting Information

Primary human pancreatic cancer cells cultivation in microfluidic hydrogel microcapsules for drug evaluation

T. Y. Song , H Zhang , Z. Q. Luo , L. R. Shang, Y. J. Zhao**

Supporting figures

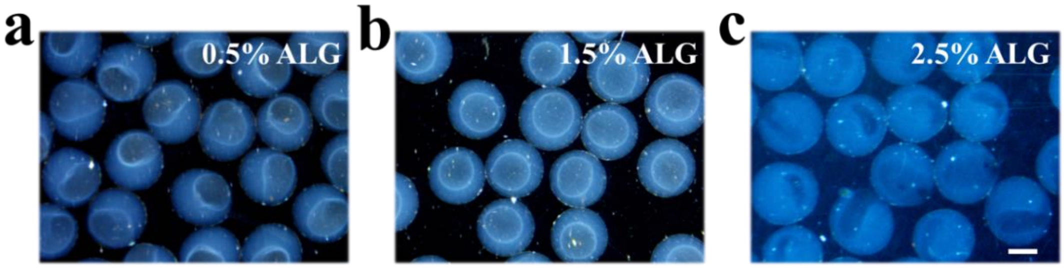

Figure S1. The change of configuration of the core-shell microcapsules by increasing the alginate concentration while keeping other parameters constant (CMC concentration: 1.0 wt%). (a) 0.5% ALG; (b)1.5% ALG; (c) 2.5% ALG. The scale bar is 200 μm.

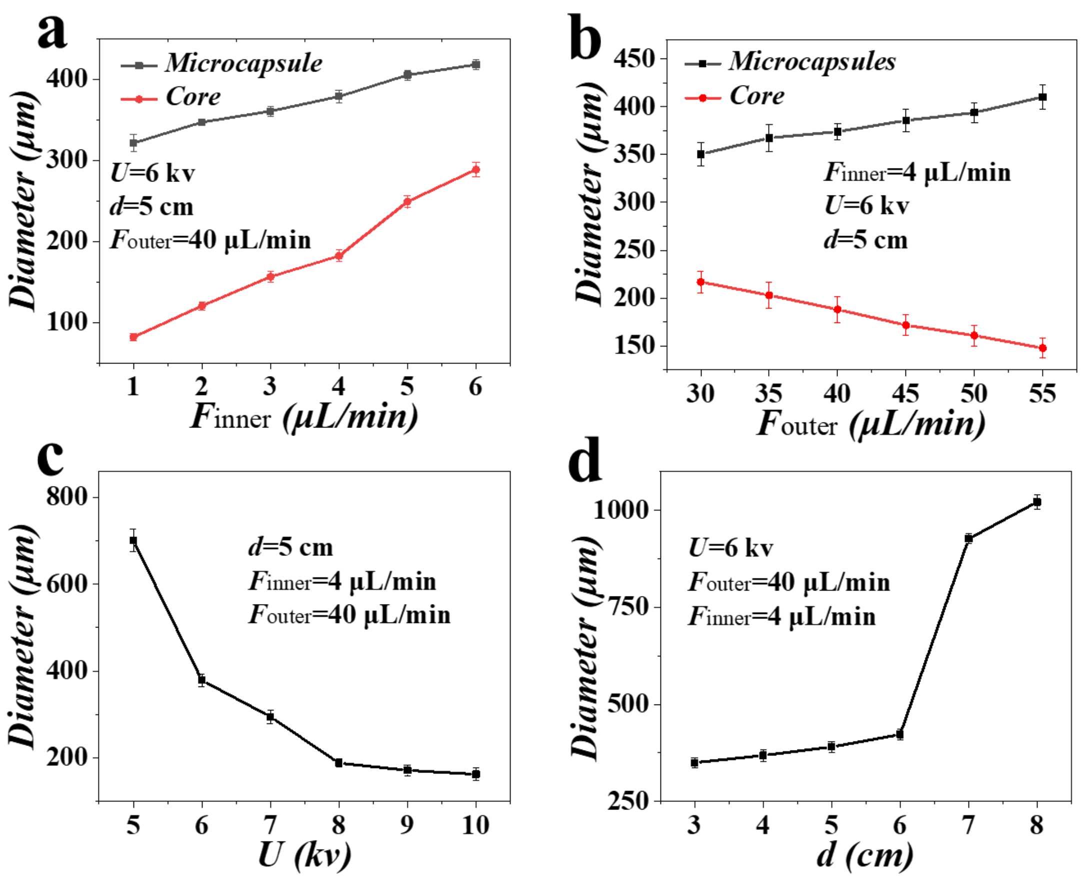

Figure S2. (a, b) The size change trend of the microcapsules and the core when adjusting (a) inner flow rate and (b) outer flow rate. (c) The size change trend of the microcapsules when increasing voltage. (d) The size of the microcapsules is a function of the collection distance. (a-d, n=50 for each group).

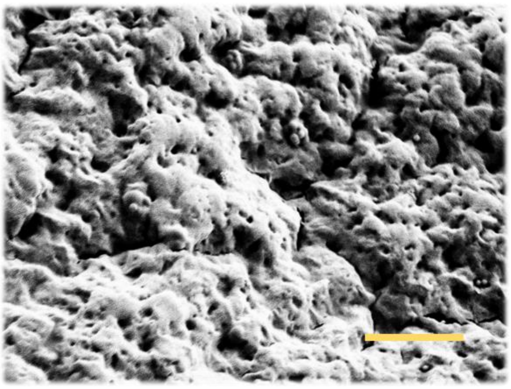

Figure S3. The porous structures of the ALG shell. The scale bar is 10 μm.

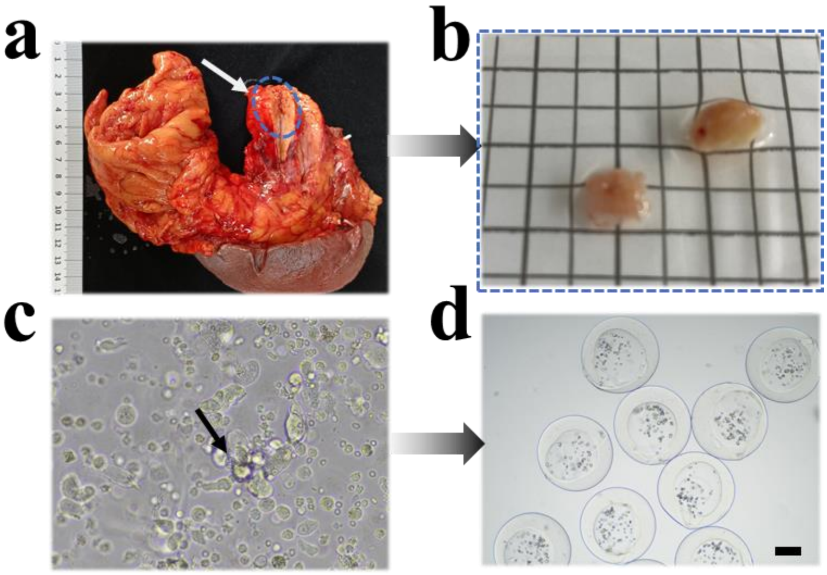

Figure S4. Gross resection of pancreatic tumor specimen, cell extraction, and encapsulation of primary tumor cells within the microcapsules. The scale bar is 100 μm.

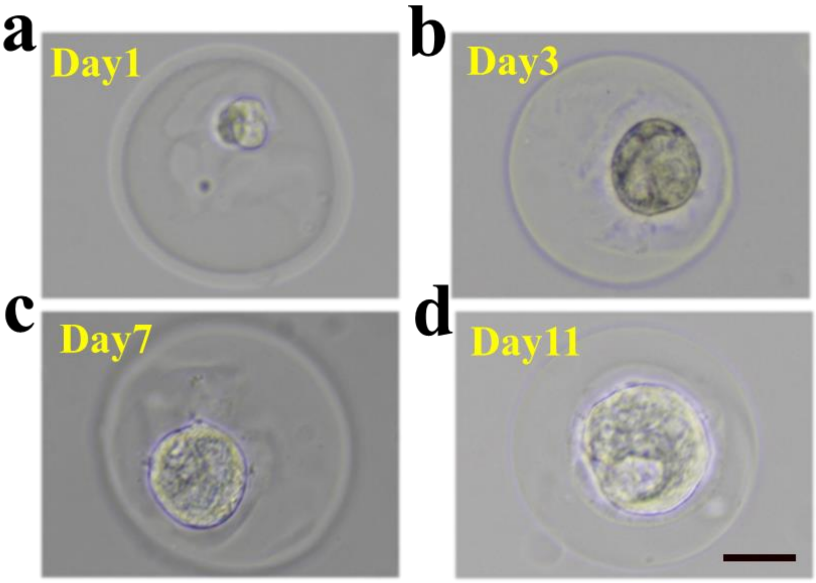

Figure S5. Bright-field images showing the proliferation of pancreatic tumor cells isolated from tumor spheroids after re-encapsulation in a hydrogel microcapsule for 1, 3, 9 and 11 days. The scale bar is 100 μm.

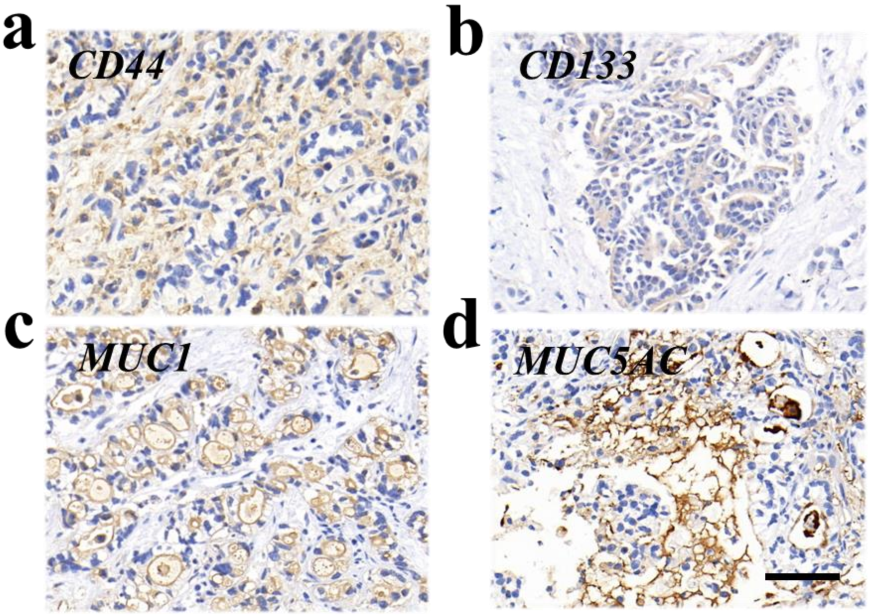

Figure S6. Immunohistochemical images of pancreatic tumor tissues for CD44, CD133, MUC1 and MUC5AC. The scale bar is 50 μm.

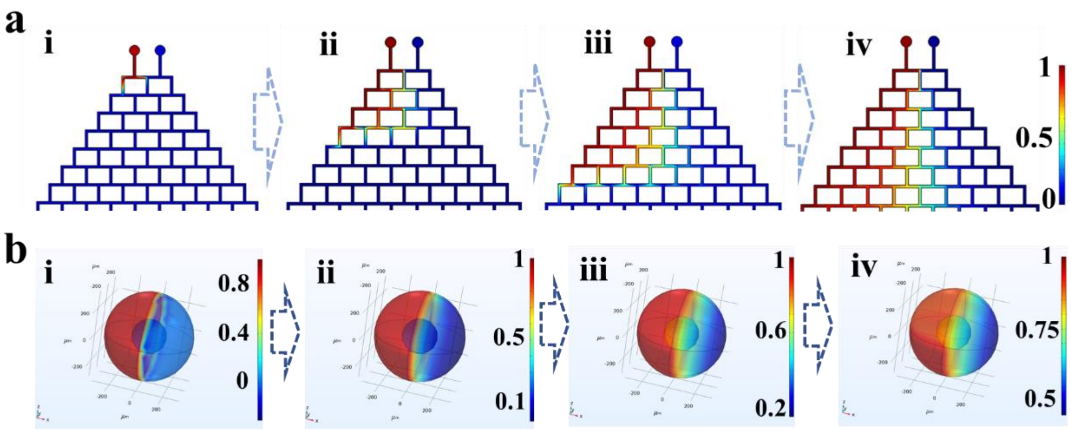

Figure S7. (a) Numerical simulation of the concentration gradient formed in the microfluidic chip. Initial drug concentration of the left and right injection fluid was set as 1.0 and 0 mol/m^3; both inlet liquid flow rate was set to 0.1 μL/min; i-iv represent the concentration gradient forming process. (b) Numerical simulation of drug permeating into microcapsules placed in the C10 culture chamber and the drug distribution under the influence of liquid flows, i-iv images represent the drug penetration process.

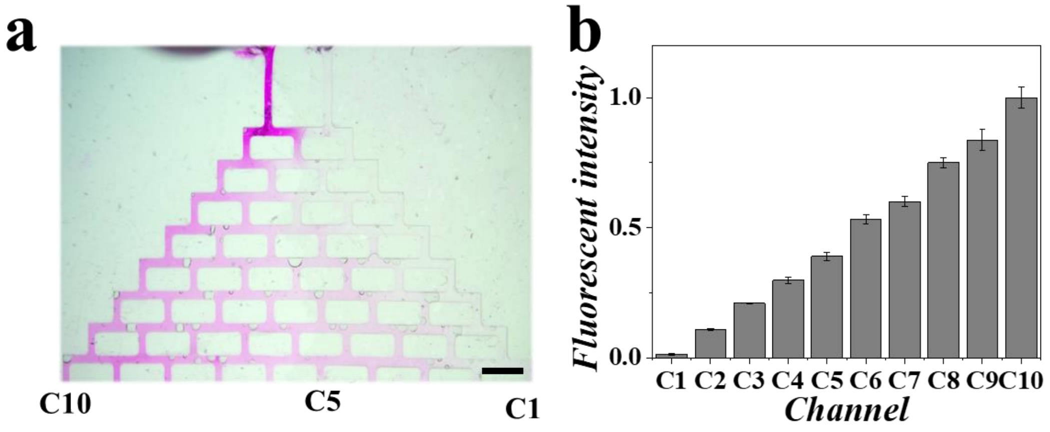

Figure S8. (a) Optical microscopic image showing Rhodamine B distribution in the microchannels of the microfluidic chip (100 μM Rhodamine fluorescence solution was pumped through the left inlet with 0.1 μL/min flow rate, and PBS solution was pumped through the right inlet with the same flow rate). (b) The fluorescent intensity of Rhodamine B was measured at the terminal branches of the microfluidic channel C1 to C10 (n=5 for each group). Data were normalized. The scale bar is 2 mm in (a).

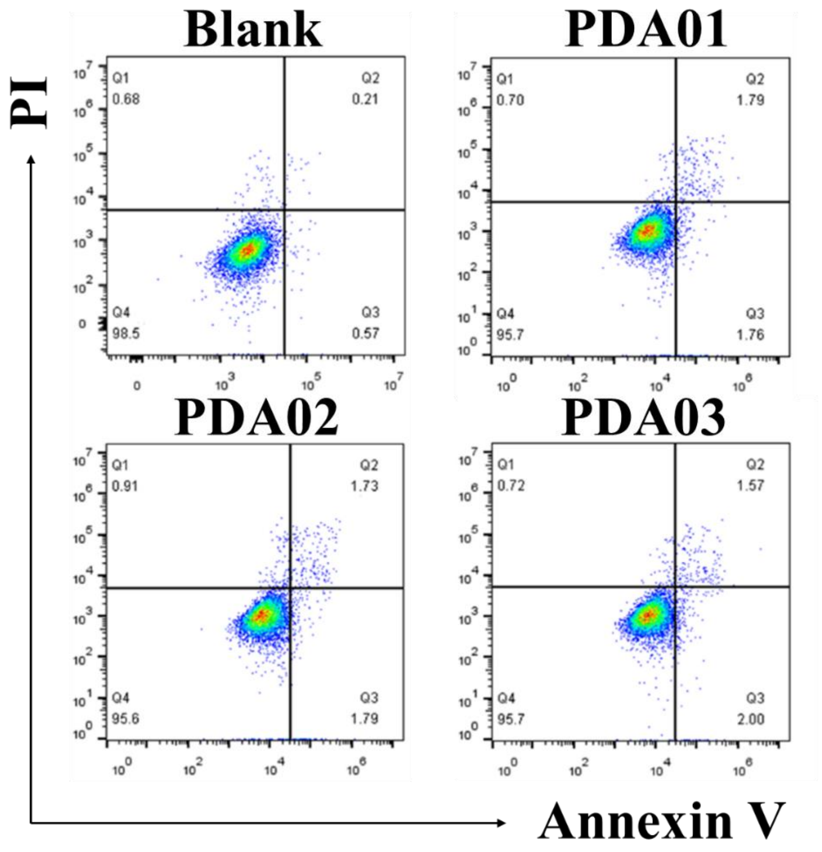

Figure S9. The cell apoptosis level of three patients-derived tumor spheroids before drug evaluation by fluorescence-activated flow cytometry.

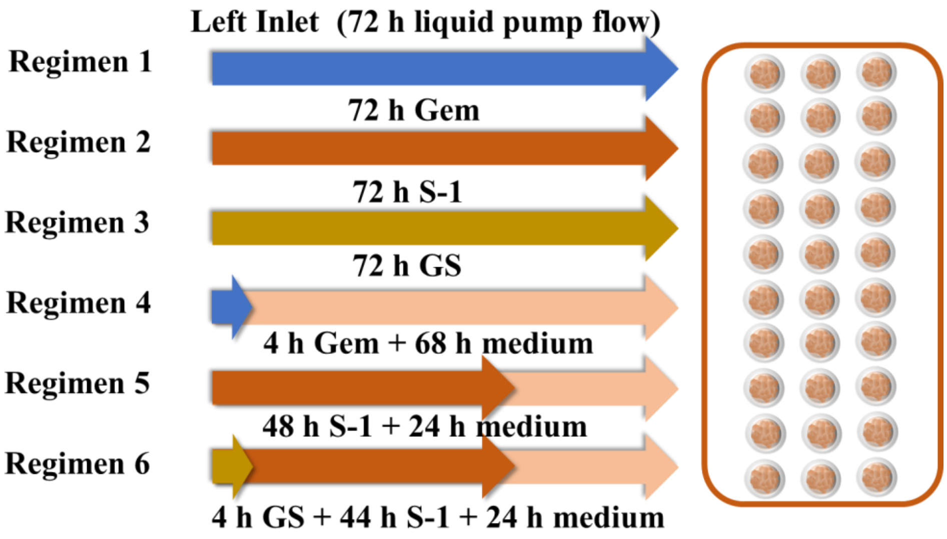

Figure S10. Schematic of the chemotherapy drugs (Gem, S-1) delivery system. Different treatment regimens were applied, including intermittent and continuous drug flows that simulate clinical drug treatment. Gem was indicated in blue, S-1 in orange, Gem and S-1 mixture in brown, and culture medium in light orange. The proportion of the colors in each row represents the treatment time of each drug. (72h Gem, 72h S-1, 72h GS, 4h Gem+68h medium, 48h S-1+24h medium, 4h GS+44h S-1+24h medium).

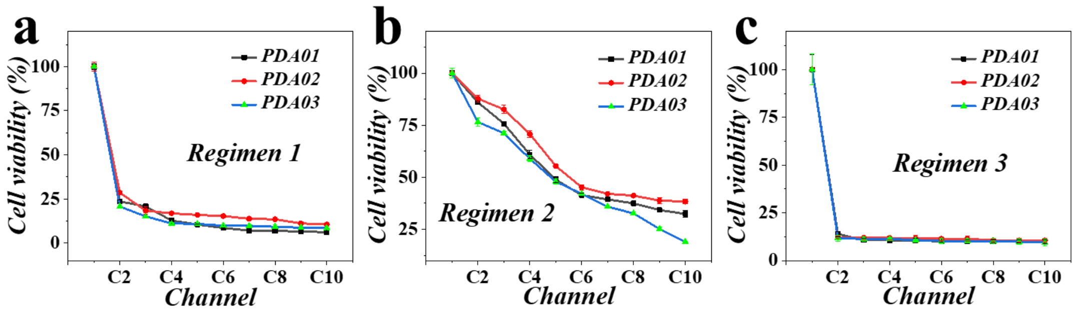

Figure S11. (a) Tumor spheroids viability in three groups over a 72-h period of continuous treatment of Gem (Regimen 1). (b) Tumor spheroids viability in three groups over a 72-h period of continuous treatment of S-1 (Regimen 2). (c) Tumor spheroids viability in three groups over a 72-h period of continuous treatment of both Gem and S-1 (Regimen 3). (a-c, n=30 for each group).
